# Editorial: Creativity and Innovation in Times of Crisis (COVID-19)

**DOI:** 10.3389/fpsyg.2022.858907

**Published:** 2022-03-23

**Authors:** Min Tang, Roni Reiter-Palmon, Zorana Ivcevic

**Affiliations:** ^1^Institute for Creativity and Innovation, University of Applied Management, Ismaning, Germany; ^2^Department of Psychology, University of Nebraska Omaha, Omaha, NE, United States; ^3^Yale Center for Emotional Intelligence, Yale University, New Haven, CT, United States

**Keywords:** creativity, innovation, COVID-19 pandemic, coping, wellbeing, theories on crisis and creativity

## Introduction

The outbreak of the COVID-19 pandemic in early 2020 has brought the world society, economy and people's daily lives into a crisis. At the time we are writing the editorial, this crisis has been accompanying us for almost 2 years and will still have far-reaching consequences beyond the spread of the disease. The focus of the current Research Topic is the effect of the COVID-19 crisis on creativity and innovation and vice versa, as well as their relationship to resilience and coping.

We are pleased to have received many submissions from authors representing different disciplines and countries. Through rigorous reviews, 34 articles contributed by 131 authors from 12 different countries were accepted. Articles include both theoretical papers and empirical studies involving samples from almost one hundred different countries and regions. These papers discuss a variety of aspects of creativity and innovation under the COVID-19 crisis situations which can be categorized into three major themes:

- The impact of COVID-19 pandemic on creativity and innovation
- The role of creativity in dealing with COVID-19
- Creative and innovative responses in times of COVID-19

In this Editorial, we give a brief introduction to the papers around these three themes. Readers are recommended to refer to the original papers to obtain more details about the wonderful theories or studies.

## The Impact of COVID-19 Pandemic on Creativity and InnovatioN

These papers treat creativity and innovation as dependent variables and investigate whether people are more, the same, or less creative in times of crisis. Furthermore, some studies also examine the social or cultural attributes of creativity/innovation during COVID-19.

### COVID-19 Pandemic and Creativity

Studies from several countries have provided empirical evidence for an increase in instances of creativity. Mercier et al. discovered a significant increase in everyday creativity (but not professional creativity) during the lockdown period among over 1,000 French participants. They also found that everyday creative behavior increased more for participants with a lower baseline creativity (before lockdown) than those with a higher baseline creativity. Hofreiter et al. echoed the findings of Mercier et al. by reporting a significant increase in almost all everyday creativity activities investigated in both Chinese and German samples. This study also explored the role of emotions and motivation on creative activities. Karwowski et al.'s diary studies revealed that Polish college students engaged in slightly, yet statistically significantly more creative activities during the lockdown than before the onset of the COVID-19 pandemic. It was also found that the more the students were engaged in discussing or searching for information about COVID-19, the more they reported taking on creative activities. With data from 74 different countries, Morse et al. reconfirmed people's increased engagement in creative leisure activities during early COVID-19 times.

### Sociocultural Impact on Creativity

These papers provided a closer look into the social or cultural factors that may shape the pandemic-creativity/innovation link. Shi et al. examined the role of family support on children's creativity and discovered the facilitating effect of family support on children's originality in creative drawing through increased perseverance in information searching. Anderson et al.'s mixed methods study of American school teachers' creativity and wellbeing during the COVID-19 pandemic emphasized the importance of environmental support and encouragement for creativity in school for teacher's wellbeing. Through a large-scale cross-cultural study involving 84 countries, Kapoor et al. analyzed the COVID-19 lockdown stringency and culture-innovation relationships and found that regardless of the degree of the stringency of the government, more collectivist and higher power-distance countries showed lower innovation. In more individualistic and lower power-distance nations, higher innovation was associated with less stringency of the government, whereas lower innovation was associated with more stringency.

## The Role of Creativity in Dealing With COVID-19

Half of the studies in the Research Topic treat creativity and innovation as independent variables or covariates and examine how creativity and innovation help people cope with the stress and challenges posed by the pandemic. Both theoretical and empirical studies were contributed to this topic.

### Theories on Crisis and Creativity

Kapoor and Kaufman described how engaging in creativity can help people guard against the negative effects of the pandemic and how entities at the personal, community, and national levels cultivated and expressed creativity in an effort to make meaning during COVID-19. Araki and Cotellessaa emphasized the importance of the creative polymathic (broader, deeper and more integrated) approach in understanding and coping with the challenges posed by crises such as COVID-19. Finally, Beghetto discussed how crises serve as an important catalyst and impetus for creative action and innovative outcomes from an agentic perspective.

### Creativity and Coping

One of the researchers' primary concerns is whether the negative consequences of the pandemic such as stress, anxiety, and isolation may hinder people's creativity. Du et al. investigated the role of negative moods on creativity among Chinese college students and discovered that negative moods facilitated creativity during the pandemic thanks to their link to cognitive and emotional creativity. Kerr et al. examined the potential impact of the COVID-19 crisis on the growing trends in depression, anxiety, and suicidality among creative students. They found that while most American creative students they studied perceived the pandemic as having a detrimental impact on their mental health, they also reported producing a remarkable variety of creative works during that time. Patston et al. evaluated Australian secondary school students and found that they demonstrated overall positive attitudes toward their learning during the COVID-19 period, and could associate opportunities for creativity with enjoyability, subject relevance, and self-efficacy. C. Tang et al. found that Chinese employees' perceived work uncertainty triggered by COVID-19 enhanced creativity when an employee's value of Zhongyong (the middle-way thinking) is low and creative self-efficacy is high. Taken together, these studies show that despite negative psychological reactions triggered by the pandemic, both students and employees across countries continued to create in times of crisis.

### Creativity and Wellbeing

The relationship between creativity and wellbeing has become a hot topic for the researchers and several papers examined this topic from different perspectives.

#### Cross-Cultural Studies of Crisis, Creativity, and Wellbeing

M. Tang et al. examined whether the crisis-creativity-wellbeing link was stable across cultures and how culture influenced this link by comparing the models between employee samples from China, Germany, and USA. Their study confirmed the hypothesized link in relation to flourishing wellbeing across the three samples. They also found that in less individualistic countries like China, creativity was related to social connectedness. In a similar vein, Morse et al.'s large-scale cross-cultural study found that maintaining or increasing time spent on leisure activities predicted greater wellbeing levels during COVID-19.

#### Novelty-Seeking and Wellbeing

Li et al. examined how novelty-seeking and mental health outcomes changed among Chinese university students before, during, and after the COVID-19 pandemic lockdown. They found that at all three-time points, higher levels of novelty seeking were associated with lower levels of stress, anxiety, and depression. Liang et al. investigated the relationship between two types of novelty-seeking behavior (i.e., the novelty input and novelty output) and individual boredom state during home quarantine. They discovered that novelty output negatively predicts the boredom state for Chinese college students with high creativity, whereas novelty input positively predicts the boredom state for persons with low creativity. Zu et al.'s experimental study found that when people are faced with mortality, they value novelty more when judging creativity. Taken together, these findings highlight the crucial role of novelty-seeking behaviors and attitudes in responding to stress, anxiety, depression, and boredom in times of crisis.

#### Specific Types of Creativity and Wellbeing

Several studies examined specific types of creativity and their connections to wellbeing. Zhai et al. discovered that emotional creativity can promote posttraumatic growth and mental health during the COVID-19 pandemic through perceived social support and regulatory emotional self-efficacy. Cui et al. found that benevolent creativity could act as an anxiety buffer when mortality is salient. Through an experimental intervention, Zuo et al. confirmed that divergent thinking training can help teenagers handle their psychological difficulties during the COVID-19 pandemic by reducing anxiety and maintaining self-efficacy. Orkibi proposed the concept of creative adaptability and demonstrated that it may buffer the impact of people's concerns about COVID-19, contributing to overall wellbeing. Despite the possible favorable impact of creativity on people's wellbeing, as demonstrated in the above-mentioned research, Wang et al. warned that being creative does not guarantee wellbeing while dealing with a traumatic event. Their studies with Chinese teens revealed that emotional resilience moderates the association between creativity and intrusive rumination.

## Creative and Innovative Responses During COVID-19

### Educational and Social Innovation

Various novel educational programs and activities have sprung up in reaction to the pandemic and this Research Topic has received two studies from China dealing with this issue. Y. Li et al. designed and delivered micro-courses based on the Thinking-based Instruction Theory (TBIT). Compared to the national curriculum, these micro-courses not only improved the course quality but also enhanced students' motivation and facilitated their online learning behavior. Yu, Liu, et al.'s study of Chinese college teachers revealed that teachers' online informal learning (see H. Yu et al.) in the pandemic is positively related to their teaching efficacy, information and communication technology efficacy as well as innovation in teaching. The association between informal learning and innovative teaching performance was sequentially mediated by personal teaching efficacy and autonomous motivation.

Since the COVID-19 pandemic has interrupted agricultural production and caused famine for millions of people worldwide, Huang and Tsai used case study to review the food industry in rural China. It demonstrated how social innovation in food production and distribution might aid social development and alleviate poverty, with implications for crisis situations.

### Humor During COVID-19

Humor emerged to be a topic among some Western but not Asian researchers. This reflects the finding that humor is associated with creativity in implicit theories of lay persons in Western countries, but not in Asia (e.g., Rudowicz, 2003; Lan and Kaufman, 2012; Luescher et al., 2019). Canestrari et al. studied how humor-based coping strategies helped Italian healthcare professionals deal with stress during the pandemic. Healthcare professionals who reported more use of humor-based coping strategies perceived the situation as less stressful. Additional studies focused on cyber activities, such as memes on social media. Glaveanu and de Saint Laurent from Switzerland looked into what makes a COVID-19 meme creative and discovered that individuals valued elaboration and humor more than traditional criteria like surprise and meaningfulness. Cancelas-Ouviña studied humorous COVID-19 memes that have flooded social media in the aftermath of COVID-19 in Spain and highlighted that humorous memes used irony, ingenuity, and creativity to make a tough and stressful situation more bearable.

### Media Responses

Some studies examined the media responses to COVID-19 in relation to creativity. Jia et al. evaluated the effects of general and creative publicity (i.e., Chinese poetry) at different times during the COVID-19 outbreak in China. They observed that creative and general publicity had a distinct impact during different periods of the COVID-19 pandemic, which might be related to the publicity format and people's psychological states at the time. Wen et al. conducted two experiments to explore the influence of various types of information in news reports regarding COVID-19 on people's mental states and behavioral intentions during the COVID-19 crisis. They discovered that in the real-time priming condition, innovative form of information that included visualized graphics could enhance the impact of positive and negative information framing on the individuals' risk perception, positive emotion, and willingness to help others, compared to plain text form. Wu et al. studied the effects of internet language related to COVID-19 (CINL) on mental health of college students and discovered that CINL could alleviate college students' depression and anxiety by increasing the cognitive flexibility. Mattson et al. analyzed 50 online news articles about creativity and COVID-19 published in two American mainstream newspapers and discovered that despite the COVID-19 quarantine having forced social isolation, people have renewed and sustained creative actions and responses in various domains throughout the pandemic, such as architecture, fashion, and faith.

## Conclusion

The collection of papers in this Research Topic helps us obtain a better understanding of the *dialectical* and *reciprocal* relationship of crisis, creativity, and innovation during the initial phase of the unprecedented COVID-19 pandemic. On the one hand, COVID-19 triggers creativity and innovation in that the novel, unknown situations and new challenges COVID-19 poses drives people to think out of the box and try new things. On the other hand, creativity and innovation help people make meaning of the unknown circumstances, cope with negative consequences and achieve personal and social wellbeing. In addition, we also find that creativity seems to be an integral part of people's reactions to crisis, be it in the form of adapted way of learning or teaching, humor, or media responses.

This Research Topic makes unique contributions to the sparse literature on the relationship of crisis, creativity and innovation. The articles add evidence to the positive hidden potential of creativity (Richards, 2007; Kaufman, 2018), particularly in times of crisis, thus having profound implications for crisis management, change management, coping mechanism, individual and social wellbeing, etc.

We thank all authors who have made excellent contribution to help us obtain this understanding. We also thank the reviewers for their efforts to guarantee the high quality of the papers. We hope that this Research Topic will attract more research attention to this topic, thus helping more individuals, organizations, and societies better cope with crises through creativity and innovation.

## Author Contributions

MT drafted the Editorial and proposed the structure of the eBook (based on this Research Topic). RR-P and ZI revised the editorial and the structure. All authors contributed to the article and approved the submitted version.

## Conflict of Interest

The authors declare that the research was conducted in the absence of any commercial or financial relationships that could be construed as a potential conflict of interest.

## Publisher's Note

All claims expressed in this article are solely those of the authors and do not necessarily represent those of their affiliated organizations, or those of the publisher, the editors and the reviewers. Any product that may be evaluated in this article, or claim that may be made by its manufacturer, is not guaranteed or endorsed by the publisher.
